# Purulent Pericarditis With Cardiac Tamponade Triggered by Left-Sided Infective Endocarditis in Active Systemic Lupus Erythematosus: A Case Report

**DOI:** 10.7759/cureus.102897

**Published:** 2026-02-03

**Authors:** Shunki Hiyama, Taichi Kato, Kazuhiro Sugiyama

**Affiliations:** 1 Tertiary Emergency Medical Center, Tokyo Metropolitan Bokutoh Hospital, Tokyo, JPN

**Keywords:** cardiac tamponade, infective endocarditis, intracerebral hemorrhage, mitral leaflet perforation, pericardial drainage, purulent pericarditis, septic shock, staphylococcus aureus bacteremia, systemic lupus erythematosus

## Abstract

Systemic lupus erythematosus (SLE)-associated pericardial effusion is generally sterile, and purulent pericarditis is rare; however, delayed recognition can be fatal. In active SLE, immunologic abnormalities - such as hypocomplementemia and impaired neutrophil function - together with concurrent immunosuppressive therapy, may predispose patients to invasive infections. We report a rare clinical course in which left-sided infective endocarditis in a patient with active SLE progressed to purulent pericarditis and cardiac tamponade, underscoring the need for rapid diagnostic consideration and timely intervention. A 32-year-old woman presented with fever, dyspnea, and rapidly progressive shock. Imaging revealed massive circumferential pericardial effusion with cardiac compression. After obtaining blood cultures, empiric intravenous antimicrobial therapy was initiated, and fluoroscopy-guided pericardial drainage yielded a large volume of brownish, purulent-appearing effluent. Transthoracic echocardiography demonstrated vegetation and perforation of the anterior mitral leaflet with mitral regurgitation. Blood cultures grew methicillin-susceptible *Staphylococcus aureus*, and she was diagnosed with left-sided infective endocarditis complicated by purulent pericarditis. Because emergent surgical indications were absent early in the course and postoperative infectious risk was a concern in the setting of active purulent pericarditis, management prioritized infection control and hemodynamic stabilization. However, cardiac surgery became infeasible after a catastrophic intracerebral hemorrhage, and the patient died on hospital day 38. This case highlights the key clinical takeaway that, in patients with active SLE and pericardial effusion accompanied by fever or hemodynamic deterioration, clinicians should promptly evaluate for infectious pericarditis and concomitant infective endocarditis by obtaining blood cultures, performing echocardiography for valvular involvement, and, when indicated, undertaking timely pericardial drainage with microbiological evaluation.

## Introduction

Systemic lupus erythematosus (SLE) is a prototypical autoimmune disease characterized by abnormalities in both innate and adaptive immunity, leading to relapsing-remitting disease activity and multi-organ involvement [1]. Cardiovascular manifestations include pericarditis, myocarditis, valvular disease, and coronary artery disease; among these, pericarditis (including pericardial effusion) is one of the major cardiac complications of SLE. During active SLE, serositis occurs with a certain frequency, with cohort studies reporting serositis in approximately 16% of patients; however, most cases are immune-mediated and sterile [2,3].

Although *Staphylococcus aureus* bacteremia and infective endocarditis (IE) are encountered with some regularity in clinical practice, purulent pericarditis remains exceedingly rare even today, and the incidence of bacterial (purulent) pericarditis in the modern antibiotic era has been estimated at approximately one in 18,000 [4]. Nevertheless, it is a life-threatening condition that can rapidly progress to cardiac tamponade and septic shock, and prognosis depends on prompt diagnosis and timely therapeutic intervention, including pericardial drainage and appropriate antimicrobial therapy. The presence of bacteremia, including *S. aureus*, and an identifiable infectious focus are key considerations in its pathogenesis.

IE is characterized by vegetation formation on cardiac valves and perivalvular abscesses and can result in severe complications such as acute valvular dysfunction due to valve destruction, heart failure, and embolic events [5]. *S. aureus* is among the leading causative pathogens, and when accompanied by bacteremia, evaluation with IE in mind is essential [6].

Here, we report a case in which active SLE was complicated by methicillin-susceptible *S. aureus* (MSSA) bacteremia; left-sided IE precipitated purulent pericarditis (purulent pericardial effusion) and cardiac tamponade. We also discuss disease mechanisms in the context of underlying SLE, diagnostic pitfalls, and challenges in management strategies, including the timing of surgical intervention.

## Case presentation

A 32-year-old woman originally from Southeast Asia was brought to our emergency department after her friend reported progressive dyspnea, followed by suspected deterioration in mental status. Four months before this presentation, she had been hospitalized at another institution for septic shock requiring mechanical ventilation and broad-spectrum antibiotics; *S. aureus* was isolated from blood cultures in the setting of pneumonia. After discharge, she was transferred to another hospital where anticoagulation with edoxaban 30 mg once daily was initiated for deep vein thrombosis (DVT), and low-dose prednisolone (5 mg/day) was started for persistent fever and a generalized rash of unclear etiology. She subsequently stayed at a rehabilitation facility for approximately two months. During that period, systemic rash, arthralgia, persistent tachycardia, and low blood pressure continued; she requested discharge home about one month before the current admission. Three weeks prior to presentation, outpatient reassessment revealed persistent fever and a markedly elevated antinuclear antibody titer (ANA: 1:2560). Hospitalization was considered; however, because of unresolved financial issues, she continued outpatient management.

On arrival, she was febrile with a mild altered mental status: Glasgow Coma Scale score of 14, temperature of 39.9°C, heart rate of 93 beats/min, blood pressure of 111/61 mmHg, respiratory rate of 36 breaths/min, and oxygen saturation of 95% on 10 L/min oxygen via a reservoir mask. She appeared severely cachectic (height: 153 cm, weight: 42 kg, body mass index: 18 kg/m²). Physical examination revealed petechial hemorrhages of the palpebral conjunctiva and a generalized polymorphous erythematous rash with dryness, scaling, and brownish discoloration. Arterial blood gas analysis showed high-anion gap metabolic acidosis with hyperlactatemia (pH: 7.26, PaCO₂: 34 mmHg, HCO₃⁻: 12.3 mmol/L, lactate: 6.2 mmol/L). Initial laboratory testing also demonstrated marked inflammation (C-reactive protein (CRP): 23.29 mg/dL), renal dysfunction, and coagulopathy. Laboratory findings at the time of hospital admission are summarized in Table 1.

**Table 1:** Laboratory findings on admission

Test	Result	Lower limit	Upper limit	Unit
White blood cell count	9.4	3.3	8.6	×10^9^/L
Hemoglobin	6.7	11.6	14.8	g/dL
Platelet count	170	130	350	×10^9^/L
Prothrombin time-international normalized ratio	1.03	0.9	1.1	INR
Activated partial thromboplastin time	33.5	24	39	sec
Fibrinogen	275	200	400	mg/dL
D-dimer	75.6		<1.0	μg/mL
Fibrin/fibrinogen degradation products	147.3		<5.0	μg/mL
Antithrombin activity	69.5	80	120	%
Total protein	7.8	6.6	8.1	g/dL
Albumin	2.1	4.1	5.1	g/dL
Blood urea nitrogen	81.2	8	20	mg/dL
Creatinine	1.76	0.46	0.79	mg/dL
Total bilirubin	0.67	0.4	1.5	mg/dL
Sodium	145	138	145	mmol/L
Chloride	111	101	108	mmol/L
Potassium	5.4	3.6	4.8	mmol/L
Calcium	8.4	8.8	10.1	mg/dL
Phosphate	5.2	2.7	4.6	mg/dL
Creatine kinase	512	41	153	U/L
Aspartate aminotransferase	54	13	30	U/L
Alanine aminotransferase	15	7	23	U/L
Lactate dehydrogenase	573	124	222	U/L
Alkaline phosphatase	47	38	113	U/L
Gamma-glutamyl transferase	30	9	32	U/L
Glucose	125	73	109	mg/dL
HbA1c	6.6	4.9	6	%
C-reactive protein	23.29	0	0.14	mg/dL

Emergency management (hospital day zero)

**Figure 1:**
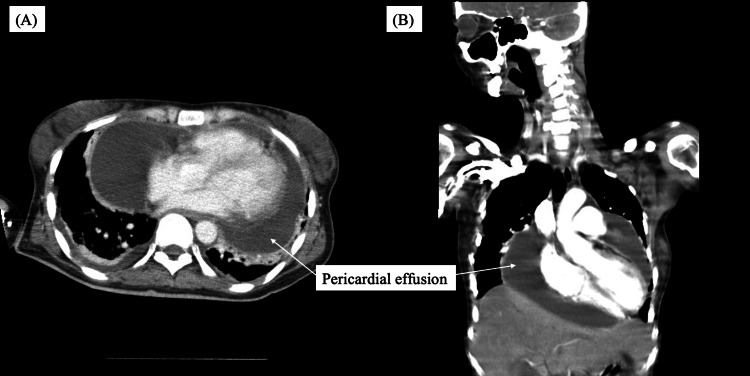
Chest computed tomography on day 0 showing pericardial effusion (A) Axial view showing circumferential pericardial effusion. (B) Coronal view confirming extensive pericardial effusion compressing the heart.

Her hemodynamic status deteriorated rapidly, with progressive circulatory failure (systolic blood pressure in the 80s mmHg, with a mean arterial pressure of approximately 60 mmHg) and tachycardic trend (heart rate increased to 140 beats/min). Point-of-care ultrasonography and contrast-enhanced computed tomography demonstrated massive circumferential pericardial effusion with cardiac compression (Figure 1).

She was diagnosed with mixed shock due to septic shock and obstructive shock from cardiac tamponade. After obtaining blood and other relevant cultures, empiric antimicrobials with vancomycin plus meropenem were initiated (meropenem administered as extended infusion, total daily dose: 3 g) to ensure early broad-spectrum coverage and optimize pharmacodynamic target attainment in the setting of septic shock. Fluoroscopy-guided pericardial drainage catheter placement yielded a large volume of brownish, purulent-appearing effluent (Table 2).

**Table 2:** Pericardial fluid analysis LDH: lactate dehydrogenase

Parameter	Result	Unit
Appearance	Purulent	
Total nucleated cell count	72,534	cells/µL
Differential – polymorphonuclear cells	68,267 (94.1%)	cells/µL
Differential – mononuclear cells	4267	cells/µL
Differential – other cells	4800	cells/µL
Specific gravity	1.041	
Osmolality	325	mOsm/kg
Total protein	5.8	g/dL
LDH	4528	U/L
Glucose	2	mg/dL
Culture	Staphylococcus aureus	

The pericardial fluid demonstrated neutrophil-predominant intense inflammation with low glucose and elevated LDH, findings consistent with purulent pericarditis. Because of progressive respiratory failure and declining consciousness, rapid sequence intubation was performed, and mechanical ventilation was initiated. Vasopressor support with norepinephrine and vasopressin was required (maximum doses: 0.71 µg/kg/min and 2 U/hour, respectively). Inotropic support with dobutamine was started for suspected sepsis-induced myocardial dysfunction (maximum dose: 5.97 µg/kg/min), and epinephrine was transiently added for refractory instability (maximum dose: 0.05 µg/kg/min). Continuous landiolol infusion was initiated for persistent tachycardia (maximum dose: 5.95 µg/kg/min). Stress-dose hydrocortisone was administered (total daily dose: 200 mg).

Transthoracic echocardiography revealed vegetation and perforation of the anterior mitral leaflet with mild mitral regurgitation. The pericardial fluid was grossly purulent. Laboratory evaluation and systemic findings showed cytopenias, hypocomplementemia, and high-titer ANA, fulfilling classification criteria for SLE (Table 3).

**Table 3:** Autoantibody profiles and complement levels in systemic lupus erythematosus

Test	Result	Lower limit	Upper limit	Unit
Antinuclear antibody titer	1:2560		1:40	titer
Anti-SSA/Ro antibody (FEIA)	135.5		7	U/mL
Anti-SSB/La antibody (FEIA)	2.4		7	U/mL
Anti–double-stranded DNA IgG (FEIA)	≥380.0	0		IU/mL
Anti-U1 RNP antibody (FEIA)	2.7		3.5	U/mL
Anti-Smith antibody (FEIA)	1.3		2	U/mL
Complement C3	35	73	138	mg/dL
Complement C4	7	11	31	mg/dL

Based on these findings, she was diagnosed with IE complicated by purulent pericarditis in the context of SLE.

Hospital course

**Figure 2:**
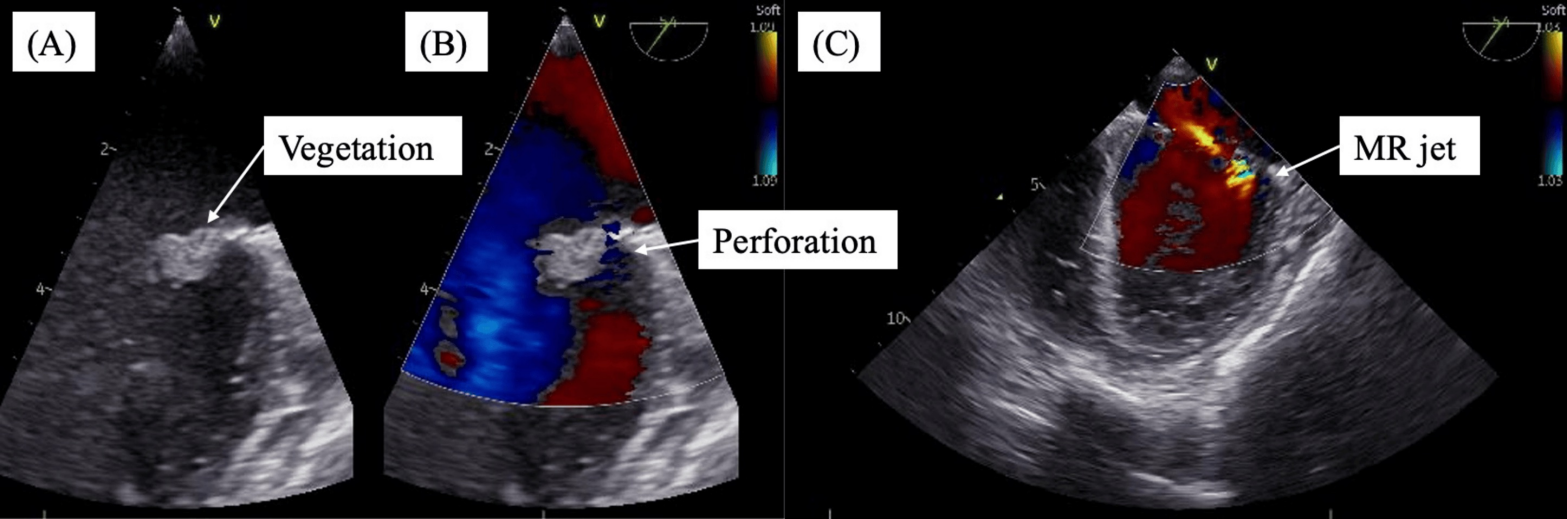
Transesophageal echocardiography on day one showing infective endocarditis (A) B-mode image showing vegetation on the anterior mitral leaflet. (B) Color Doppler image obtained in the same imaging plane demonstrating a mitral regurgitation jet through an anterior mitral leaflet perforation. (C) Additional Color Doppler view demonstrating mitral regurgitation.

On hospital day one, blood cultures grew MSSA. Antimicrobial therapy was de-escalated to ceftriaxone at a central nervous system-penetrating dose (total daily dose: 4 g) at that time, as altered mental status raised concern for possible central nervous system involvement, and this regimen was selected transiently while intracranial complications were being evaluated. Transesophageal echocardiography confirmed an approximately 10 × 5 mm vegetation on the anterior mitral leaflet with leaflet perforation and mild-to-moderate mitral regurgitation; no annular abscess was identified (Figure 2).

By hospital day three, the need for surgical intervention was discussed with the cardiothoracic surgery team. Given the anterior mitral leaflet perforation and a vegetation measuring approximately 10 mm, embolic risk was a concern, and urgent surgery could be considered according to current guideline frameworks.

However, at that time, there were no emergency indications mandating immediate surgery, such as refractory acute heart failure (e.g., cardiogenic shock or pulmonary edema). In addition, urgent indications-including uncontrolled infection (e.g., persistent bacteremia or abscess formation) or imminent/recurrent embolization-were not yet clearly established during the early phase.

Moreover, the presence of active purulent pericarditis raised concern for postoperative infectious complications associated with median sternotomy. Therefore, the initial strategy prioritized infection control and hemodynamic stabilization, with careful reassessment of surgical timing thereafter.

On hospital day four, contrast-enhanced head CT and whole-body CT showed no intracranial lesions, including infectious aneurysms. Given her preserved neurological status, antimicrobial therapy was switched from ceftriaxone to cefazolin (total daily dose: 6 g) as first-line treatment for MSSA bacteremia and IE.

**Figure 3:**
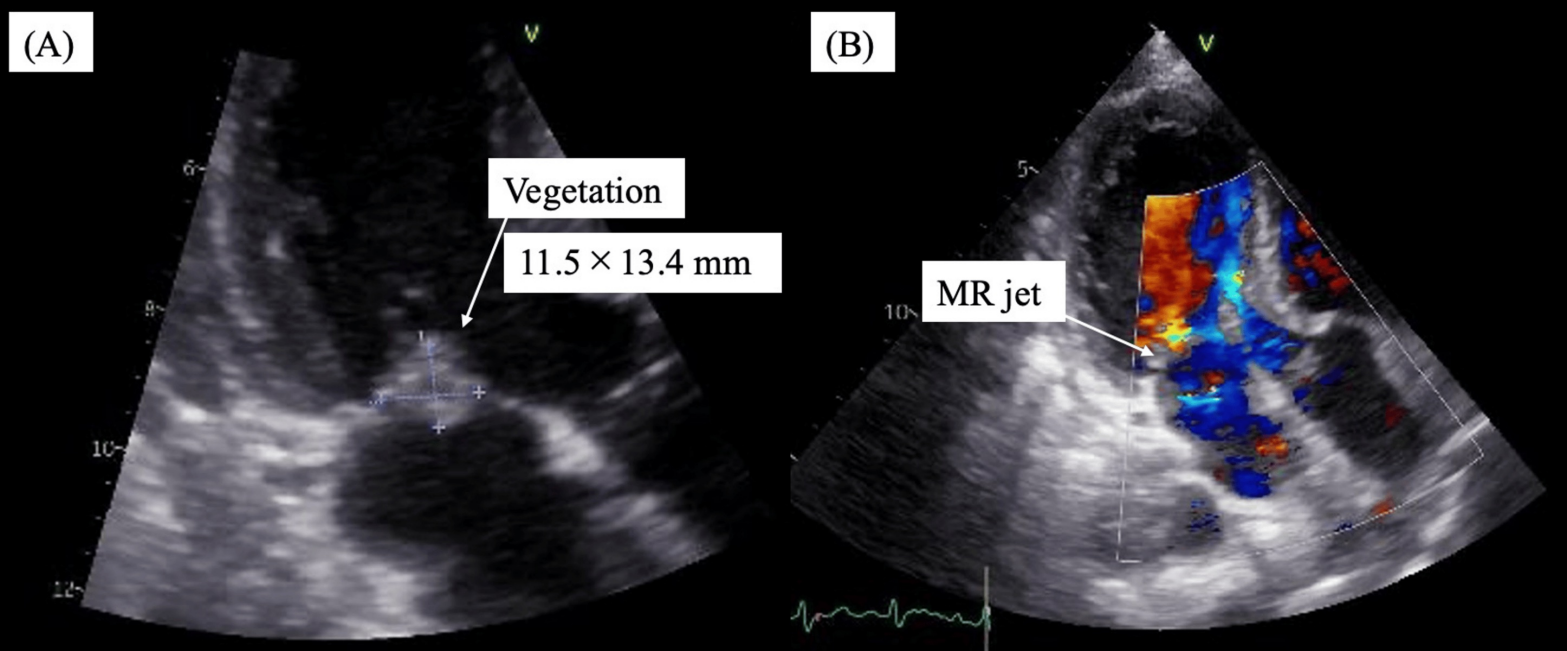
Transthoracic echocardiography on day six showing infective endocarditis (A) B-mode image showing a vegetation measuring 11.5 × 13.4 mm on the anterior mitral leaflet. (B) Color Doppler image obtained at the same imaging plane demonstrating mitral regurgitation.

On hospital day five, her hemodynamics and mental status stabilized, and inflammatory markers improved, with CRP decreasing to 4.68 mg/dL; she was successfully extubated on the same day. On hospital day six, transthoracic echocardiography was performed in the central laboratory (Figure 3).

Blood cultures were obtained on hospital days zero, two, six, seven, and nine, and blood culture clearance was ultimately confirmed with negative cultures on hospital day nine.

However, on hospital day seven, she developed recurrent high-grade fever (nearly 40°C) with shivering, and the inflammatory markers rose again, with CRP increasing to 17.54 mg/dL, suggesting inadequate infection control. For further source evaluation, contrast-enhanced trunk CT was performed, but no new infectious focus was identified. On hospital day eight, based on the repeat transthoracic echocardiography findings from hospital day six, together with concern for inadequate source control and persistent bacteremia, the case was re-discussed with the cardiothoracic surgery team, and an urgent surgical strategy was being considered.

**Figure 4:**
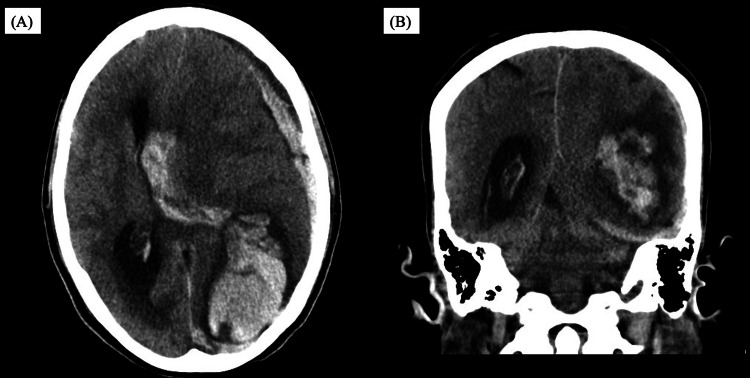
Head computed tomography on day 10 showing intracranial hemorrhage (A) Axial view showing a large left occipital intracerebral hemorrhage with intraventricular extension. (B) Coronal view confirming extensive intracerebral hemorrhage with ventricular involvement.

However, before surgical intervention could be undertaken, she developed abrupt neurological deterioration with anisocoria on hospital day 10. Head CT demonstrated a large left occipital intracerebral hemorrhage with intraventricular extension. Following a multidisciplinary discussion, cardiac surgery was deferred for at least four weeks in accordance with current guideline recommendations for IE complicated by major intracranial hemorrhage (Figure 4).

As her hemodynamics did not mandate immediate surgical intervention and there was no evidence of uncontrolled infection at that time, conservative management was continued.

Although hemodynamics remained stable and inflammatory markers improved under continued antimicrobial therapy, her overall condition gradually worsened, including progressive malnutrition and frailty. She died on hospital day 38. A summary timeline of the clinical course is provided in Table 4.

**Table 4:** Summary timeline of the clinical course

Time point	Key event
4 months before admission	Septic shock with Staphylococcus aureus bacteremia (pneumonia) at another institution.
3 weeks before admission	Persistent fever; ANA 1:2560 on outpatient reassessment.
Hospital day 0	Presentation with fever, dyspnea, and shock; imaging showed massive circumferential pericardial effusion with tamponade; pericardial drainage yielded purulent fluid; empiric antimicrobials initiated; intubation and vasoactive support required.
Hospital day 1	Blood cultures grew MSSA; antimicrobials de-escalated to ceftriaxone; TEE confirmed mitral valve vegetation with anterior leaflet perforation; no annular/perivalvular abscess.
Hospital day 3	Cardiothoracic surgery consulted; surgical timing planned for reassessment after stabilization and infection control given active purulent pericarditis.
Hospital day 4	Contrast-enhanced head and whole-body CT showed no intracranial lesions (including infectious aneurysms); antimicrobials switched to cefazolin as first-line therapy for MSSA IE/bacteremia.
Hospital day 5	Hemodynamics and mental status stabilized; inflammatory markers improved (CRP 4.68 mg/dL); extubated.
Hospital day 6	TTE performed in the central laboratory (Figure 3).
Hospital day 7	Recurrent high-grade fever with shivering (CRP 17.54 mg/dL); contrast-enhanced trunk CT performed for source evaluation but no new infectious focus identified.
Hospital day 8	Based on repeat TTE findings and concern for inadequate source control/persistent bacteremia, the case was re-discussed with the cardiothoracic surgery team and an urgent surgical strategy was being considered.
Hospital day 9	Blood culture clearance confirmed with negative cultures.
Hospital day 10	Sudden neurologic deterioration; head CT showed intracerebral hemorrhage with intraventricular extension; cardiac surgery deferred for at least 4 weeks per guidance.
Hospital day 38	Death.

## Discussion

The most distinctive feature of this case is that active SLE was complicated by MSSA bacteremia and, triggered by left-sided IE, progressed to purulent pericarditis (purulent pericardial effusion) with cardiac tamponade. Although pericarditis itself is not uncommon in patients with SLE, it is usually a sterile, immune-mediated serositis, and the development of purulent pericarditis is exceedingly rare.

This rarity may be explained by the fact that the pericardial space is generally unlikely to become a target of hematogenous seeding and, in the modern era, early initiation of antibiotic therapy often controls severe bacteremia before it can extend to the pericardial cavity [7]. Accordingly, the establishment of purulent pericarditis likely requires the concurrence of (i) severe and persistent bacteremia and (ii) a susceptible pericardial milieu (e.g., inflamed and damaged pericardium, pre-existing pericardial effusion, or impaired local host defenses).

During active SLE, pericarditis as a manifestation of serositis is associated with increased vascular permeability and exudation, predisposing to pericardial effusion; while such effusions are typically sterile, a “closed cavity containing fluid” may become an environment in which invading bacteria are difficult to eradicate once introduced [3,8]. In addition, the immunologic features of SLE - such as complement consumption due to immune-complex formation (hypocomplementemia) and leukopenia - may impair bacterial clearance mediated by opsonization and phagocytosis [9,10]. In particular, complement depletion can weaken opsonization against organisms such as *S. aureus*, which can evade phagocytosis through capsular and surface structures, thereby rendering phagocytic killing less effective [11]. Thus, even in the presence of heightened systemic inflammation, reduced complement-dependent clearance and compromised neutrophil function may create a substrate for severe and prolonged MSSA bacteremia. When compounded by vasculitis, disruption of the skin barrier, and malnutrition, host defense may be further diminished, and the addition of corticosteroids and immunosuppressants may promote persistent bacteremia.

In this case, in addition to MSSA bacteremia, transthoracic and transesophageal echocardiography demonstrated vegetation and perforation of the anterior mitral leaflet, supporting the diagnosis of left-sided IE. In patients with SLE, Libman-Sacks endocarditis (nonbacterial thrombotic endocarditis) is an important differential diagnosis; however, the unequivocal bacteremia and overall clinical presentation in this case were consistent with the diagnostic framework of bacterial IE, and the pathophysiology was considered typical of IE [12,13]. Moreover, if SLE-associated endothelial injury and microthrombotic lesions were present on the valvular surface, they could have served as a nidus for bacterial adhesion during bacteremia, potentially facilitating the development of bacterial IE [14].

Nevertheless, we acknowledge a potential diagnostic overlap. Severe *S. aureus* endocarditis/sepsis can rarely be accompanied by autoantibody positivity and hypocomplementemia [15]. Moreover, IE with anti-double-stranded DNA antibody positivity and hypocomplementemia has been reported, with normalization of complement levels and autoantibodies after antibiotic therapy alone without immunosuppression, underscoring that infection can rarely mimic lupus-like serologies [16]. Given the patient’s prior episode of MSSA bacteremia four months earlier, a remote alternative explanation is that subacute IE may have already been evolving and subsequently extended contiguously to the pericardium. Although this possibility cannot be completely excluded, the overall clinical constellation and serologic profile fulfilled SLE classification criteria, supporting concurrent active SLE as an underlying condition.

Taken together, this case suggests that, during active SLE (with pericardial effusion and local inflammation), immunologic vulnerability such as hypocomplementemia contributed to severe bacteremia and the establishment of IE, which in turn enabled spread into the pericardial space, culminating in purulent pericarditis and cardiac tamponade. In other words, rather than an incidental coexistence of rare conditions, the course can be explained by a pathophysiologic linkage in which SLE created both a “pericardial receptacle” and impaired host defense, upon which MSSA infection progressed-an aspect that underpins the clinical value of this report.

Fever and pericardial effusion in patients with SLE are often attributed to autoimmune pericarditis; however, when accompanied by hypocomplementemia or leukopenia, failure to recognize an infectious process may allow rapid progression to cardiac tamponade and septic shock. This case highlights the importance of early blood cultures, echocardiographic assessment for vegetations and valvular destruction, and, when indicated, pericardiocentesis/pericardial drainage with submission of specimens for culture to promptly exclude purulent pericarditis, despite its rarity.

From a therapeutic standpoint, this case involved IE with mitral leaflet perforation, for which surgical treatment would be considered in the longer term. However, in the acute phase of purulent pericarditis immediately after pericardial drainage, the risk of postoperative infection related to median sternotomy (e.g., mediastinitis and sternal osteomyelitis) was a major concern [17]. At the initial stage, there was no immediate indication for emergent surgery (e.g., refractory heart failure), and we therefore prioritized medical stabilization and assessed the early response to antimicrobial therapy.

In this setting of active purulent pericarditis with concern for ongoing bacteremia, the decision to proceed with early surgery required careful risk-benefit balancing between the potential benefit of mitigating embolic risk and the heightened risk of postoperative infectious complications associated with median sternotomy. Given the initial clinical improvement and the great concern for postoperative infectious complications, we continued intensive antimicrobial therapy and supportive care.

After extubation, however, the clinical course relapsed with recurrent high-grade fever and renewed inflammatory activity, and bacteremia remained a concern. In light of the repeat transthoracic echocardiography findings (with no clear reduction in vegetation burden) and the need for source control to achieve durable bacteremia clearance, the case was re-discussed with the cardiothoracic surgery team, and an urgent surgical strategy was being considered, despite the ongoing infection-related operative risk. Nevertheless, the patient developed an intracranial hemorrhage during the waiting period. Robust evidence regarding optimal surgical timing for IE complicated by purulent pericarditis is limited, and the decision to prioritize infection control versus to accept risk and proceed with early surgery remains a highly challenging, case-specific clinical judgment.

In this case, the patient had been receiving edoxaban for DVT before admission, and it was discontinued on presentation. Although a direct causal relationship cannot be established, antecedent anticoagulation may have increased the risk of intracranial hemorrhage. With respect to neurologic complications of IE (ischemic stroke, intracranial hemorrhage, infectious aneurysm, etc.) and the timing of cardiac surgery, the European Society of Cardiology (ESC) guidelines and the American Heart Association (AHA) scientific statement generally recommend postponing surgery for approximately four weeks after major intracranial hemorrhage [18,19]. Recent large observational studies suggest that surgery within one week after ischemic stroke without hemorrhagic complications may not necessarily increase mortality; however, the risk of worsened neurologic outcomes remains a concern, underscoring the need for careful individualized decision-making [20].

Finally, in this case, inflammatory markers and systemic symptoms persisted even after septic shock, and clinical features suggestive of SLE were present; nonetheless, socioeconomic factors may have delayed adequate diagnostic evaluation and timely therapeutic intervention. In circumstances where active autoimmune disease and severe infection can mutually exacerbate one another, early diagnosis and access to inpatient treatment directly influence outcomes. Among foreign patients, language, administrative, and financial barriers may overlap; this case underscores the importance of early incorporation of medical social work and interpretation/medical navigation support to mitigate such barriers.

## Conclusions

This case illustrates a rare clinical course in which active SLE was complicated by MSSA bacteremia and subsequently progressed to purulent pericarditis and cardiac tamponade, triggered by left-sided infective endocarditis. Although pericarditis and pericardial effusion in SLE are often sterile, patients may develop invasive infections in the setting of underlying immunologic vulnerability; therefore, infectious pericarditis should not be excluded from the differential diagnosis when pericardial effusion is accompanied by fever or hemodynamic deterioration. Prompt acquisition of blood cultures, echocardiographic assessment of valvular lesions, and, when indicated, timely pericardial drainage with microbiological evaluation are essential.
